# Organisational Culture and Healthcare Workforce Performance in Vietnam: The Mediating Roles of Knowledge Sharing and Digital Transformation

**DOI:** 10.3390/healthcare14162631

**Published:** 2026-08-20

**Authors:** Ngoc Doan Thu Vuong, Vissanu Zumitzavan, Grichawat Lowatcharin

**Affiliations:** College of Public Affairs and Policy, Khon Kaen University, Khon Kaen 40002, Thailand; ngocdoanthu.v@kkumail.com (N.D.T.V.); grichawat@kku.ac.th (G.L.)

**Keywords:** digital transformation, job performance, knowledge-sharing behaviour, organisational commitment, organisational culture, healthcare, denison model, PLS-SEM, Vietnam

## Abstract

Background: Healthcare access in Vietnam confronts regional disparities and rising demand, requiring collaborative policies and strong workforce performance to guarantee effective and affordable services. This study scrutinises how organisational culture shapes healthcare workforce performance within Vietnam’s public healthcare organisations, with particular emphasis on organisational commitment, knowledge-sharing behaviour, and digital health transformation. Methods: This study employed a quantitative, cross-sectional survey design. Data were collected from 450 healthcare professionals in public healthcare facilities in southern Vietnam through convenience and snowball sampling, using a structured questionnaire measuring organisational culture (Denison model), organisational commitment, knowledge-sharing behaviour, digital health transformation and job performance on a six-point Likert scale. Data were analysed using partial least squares structural equation modelling (PLS-SEM) in SmartPLS 3.0. Results: The findings indicate that organisational culture is directly and substantively associated with healthcare workforce performance. In addition, knowledge sharing and digital health transformation operate as mediating mechanisms, with knowledge sharing showing a notably stronger indirect association. Notably, the relationship between organisational commitment and healthcare workforce performance was indicated to be not statistically significant. Conclusions: This study offers important practical and healthcare policy implications for healthcare administrators, human resource practitioners, public-sector leaders, and health policy stakeholders, predominantly in designing and employing targeted, culture-driven interventions aimed at improving healthcare workforce performance. Specifically, fostering a culture that encourages knowledge exchange and supports digital health transformation emerges as a critical pathway for organisational advancement. Such initiatives are instrumental in nurturing a motivated, engaged, and high-performing workforce, thereby underlining the sustainable development of public healthcare organisations.

## 1. Introduction

Health workforce capacity is a first-order constraint on health systems worldwide. The World Health Organisation places the global health workforce at 65.1 million in 2020 and projects a shortfall of approximately 10 million health workers by 2030, revised downwards from an earlier estimate of 18 million but distributed highly unevenly: workforce density differs 6.5-fold between high-and-low-income countries [1,2]. This global trend is starkly reflected at the national level; for instance, the Ministry of Health forecasts that for the 2021–2030 period, Vietnam’s healthcare system requires approximately 173,400 more doctors and 313,900 more nurses [3]. Because expanding the stock of health workers is slow and capital-intensive, raising the performance of the workforce already in post has become an equally consequential policy lever, particularly across lower-and middle-income countries where the deficit is concentrated. This positions the organisational conditions that shape how healthcare professionals work—how they are led, which values are enforced, whether expertise is exchanged, and how readily new technologies are adopted—as a question of health system capacity rather than of managerial preference alone.

Furthermore, healthcare access in Vietnam is characterised by regional imbalances and socioeconomic stratification, specifically between urban centres and rural provinces [4]. In addition, the demand for healthcare services whilst reaching the citizens’ expectations for the service quality and availability is fast-growing, but the government must safeguard its affordability of customers [5]. To overcome the challenges, the healthcare sector in Vietnam must have appropriate strategies and policies calling for the participation of multiple stakeholders comprising healthcare organisations. Importantly, the role of the healthcare workforce needs to be highlighted when setting the targets because their performance plays a vital role in improving the levels of effectiveness of organisations [6,7].

To optimise this performance, it is imperative to address a framework of interconnected pillars. Specifically, organisational culture is an indispensable core of any organisational activity in order to ensure all the members’ consistent implementation of policies. Organisational contexts and shared values deeply influence employees’ psychological behaviour, determining whether they remain committed and dedicated or intend to leave. For this reason, organisational culture becomes an antecedent of organisational commitment serving as a practical instrument of managerial motivation [8]. Healthcare organisations regularly require dedicated healthcare professionals, as their roles directly impact the health of patients. Thus, one of the functions of a leader is to define organisational values guaranteeing high-quality patient care. When healthcare professionals integrate these values into their work, they find their work meaningful, thereby building organisational commitment and motivating them to invest core competencies and efforts to deliver exceptional performance [9]. As a powerful force, organisational commitment motivates employees to align their actions with organisational goals. This results in higher productivity and overall effectiveness [10]. Taken together, these studies strongly suggest that investigating the connection between organisational culture, organisational commitment, and healthcare workforce performance is vital for contemporary organisations.

In addition, knowledge sharing is indispensable for organisations as it contributes to enhanced employee engagement, effective decision-making, reduced knowledge loss. Besides, it also inspires innovation and ultimately provides organisations with a competitive edge [11,12,13]. As a result, organisations have pursued numerous strategies to promote knowledge sharing, directing to enhance both individual performance and overall organisational efficiency [14,15]. Substantial empirical evidence demonstrates that knowledge-sharing behaviour among healthcare professionals is valuable. It enables medical professionals to have a meaningful exchange of expertise and experience, thereby better meeting patient needs [16]. Hence, when leaders cultivate a supportive organisational culture actively encouraging knowledge sharing, members are more inclined to engage meaningfully and participate proactively in collaborative processes. [17]. In essence, organisational culture can either facilitate or deter these processes directly influencing an organisation’s adaptability, performance, and patient care quality. Therefore, it is vital to understand and actively organise how it influences knowledge sharing to enhance healthcare workforce performance in the healthcare sector, particularly in the context of the knowledge economy and the era of digital health transformation.

Finally, the concept of digital health transformation emphasising on developing digital technologies and supporting infrastructure to create a dynamic and tech-driven model [18], appears to be essential in this study. This comprehensive approach encompasses necessary organisational change to fully integrate digitalisation as well as align with emerging digital processes and capabilities [19]. Although several studies have investigated the influence of digital health transformation on healthcare workforce performance across different fields [20,21], there remains a scarcity of empirical evidence on its effect on individual-level performance in the health sector. In particular, there persists a significant empirical gap on how organisational culture dimensions influence digital health transformation adoption in the healthcare settings. This implies that there is restricted exploration of the mediating role of digital health transformation in the association between organisational culture and healthcare workforce performance in this field.

Therefore, this study seeks to answer the following research question: To what extent does organisational culture influence the healthcare workforce performance in Vietnam’s public health sector in terms of: (a) organisational commitment, (b) knowledge-sharing behaviour, and (c) digital health transformation?

The authors begin with a literature review including the theoretical foundations of organisational culture, organisational commitment, knowledge-sharing behaviour, digital health transformation, and healthcare workforce performance informing the development of the research framework and hypotheses. The paper then details a quantitative methodology applied to collect and analyse data. Finally, the study discusses the findings in relation to the research question, highlights academic and practical implications, acknowledges research limitations, and offers recommendations for future research.

## 2. Literature Review

Based on Social Exchange Theory (SET), the relationship between an organisation and its employees relies on reciprocity; fair treatment by the organisation encourages employee dedication [22]. Research indicates that organisational commitment is fostered when organisations create a supportive cultural environment [23]. Cultivating such a strong organisational culture is essential for promoting teamwork, securing organisational commitment, and increasing employee productivity [24]. SET also explains that when organisations invest in digital transformation, employees perceive it as a commitment to innovation and their development. This investment fosters a supportive organisational culture, promoting employees to reciprocate by demonstrating higher effort to the workplace [25]. Consequently, this study utilises SET as a theoretical framework to examine the interplay between organisational culture, digital transformation, organisational commitment, and job performance in the healthcare sector.

The Theory of Planned Behaviour (TPB) predicts intentional behaviours based on subjective norms, attitudes, and perceived behavioural control [26]. In organisational contexts, TPB is widely used to evaluate employees’ perceptions and attitudes, which lead to knowledge-sharing behaviours [27]. In this manner, several studies have shown that cultural factors within organisations can impact knowledge-sharing behaviour, which effectively promotes employees’ job performance [28,29]. Therefore, this study uses the TPB framework to demonstrate how cultural elements nurture knowledge sharing and foster employee performance in healthcare settings.

### 2.1. Organisational Culture and Job Performance

Organisational culture (OCU) refers to a system of values, norms, interpretations and beliefs shared throughout the entire organisation leading or steering the behaviour of individuals and groups, distinguishes the organisation from other organisations and forms the foundation of the organisation’s identity [30,31,32,33,34].

In addition to Schein’s [35] three-level model and Cameron & Quinn’s [36] Competing Values Framework, Denison et al.’s [37] has emerged as a prevalent dimensional model [38]. Based on this perspective, organisational culture encompasses different aspects of organisational life such as mission, consistency, involvement, and adaptability, which balances internal and external focus areas as well as flexibility and stability. As a result, highly effective organisations are those that achieve high levels in all four cultural characteristics [39,40,41].

Job performance (JP) is related to an employee’s ability in accomplishing specific goals to achieve their productivity and overall effectiveness. Additionally, it reflects an employee’s behaviours toward broader support of the organisation and coworkers. Thus, healthcare workforce performance encompasses both core responsibilities and organisational citizenship behaviours [42,43,44].

Mission of an organisation serves as a strategic foundation. Employees perform better when they possess a clear understanding of objectives and are motivated by how their specific roles contribute to the organisations’ broader strategic goals [45,46]. Additionally, structural empowerment comprising access to resources, support, and opportunities, motivates nurses to improve their professional outcomes. This is directly associated with employee effectiveness and productivity in the healthcare sector [47] and enables staff to contribute more effectively to overall performance within a learning-oriented culture [48]. Furthermore, a strong research culture promotes research engagement, flexibility, and collaborative communication. These elements are vital for ongoing professional development and enable health professionals to navigate evolving patient demands, address medical challenges, and meet performance standards in dynamic environments [49,50].

As noted by Nguyen et al. [51], Denison has made great efforts in formulating a comprehensive theoretical model of organisational culture based on the characteristics of organisations. This theory also enlightens how organisational culture creates a favourable environment for employees to provide customers with high quality service [52]. Given these considerations, the present study hypothesises that:

**H1.**  *Organisational culture is positively associated with healthcare workforce performance.*

### 2.2. Organisational Culture, Organisational Commitment and Job Performance

Organisational commitment (OC) is a fundamental concept in organisational behaviour and human resources management which refers to the psychological attachment and emotional bond that employees develop towards their organisation [30]. Organisational commitment is characterised by three aspects: a strong belief in and acceptance of the organisation’s goals and values; a willingness to exert considerable effort on behalf of the organisation; and a strong desire to remain a member of the organisation [53]. The antecedents and consequences of organisational commitment have been explored transgenerationally in various contexts. It can be categorised into three groups of antecedents that affect organisational commitment: personal factors [54], job-related factors [55], and organisational factors [56]. The consequences of organisational commitment have been found under various perspectives, including performance [57].

From the organisational standpoint, organisational commitment plays a mediating role in several relationships across various contexts, such as between training and development or career development and work performance [58]; organisational learning and nurse performance [59]; organisational constraints and extra-role performance [60]. Recent studies have established the mediating role of organisational commitment between cultural aspects such as team culture, training culture, and individual work performance [49,61].

Thus, further exploration to understand the mediation of organisational commitment in a healthcare setting is essential. The following hypotheses are proposed:

**H2a.**  *Organisational culture is positively associated with organisational commitment.*

**H2b.**  *Organisational commitment is positively associated with healthcare workforce performance.*

**H2c.**  *Organisational commitment mediates the association between organisational culture and healthcare workforce performance.*

### 2.3. Organisational Culture, Knowledge-Sharing Behaviour, and Job Performance

Knowledge-sharing behaviour (KSB) is defined as a series of actions in which employees in organisations share and exchange knowledge, suggestions, ideas, experiences, and skills to enrich the expertise and best practices of their professional staff [62]. Several studies have thoroughly examined the key factors connected to the individual, organisational, and technological aspects that influence knowledge-sharing behaviours within organisations [63]. This is especially important in the healthcare context of developing countries [64].

Furthermore, the inherent culture of a team directly enhances members’ efficiency and willingness to engage in knowledge sharing, leading to positive impacts on overall team performance [29,65]. Research indicates that a robust organisational culture links to organisation’s innovation performance by facilitating knowledge sharing among employees, which enables employees to acquire the knowledge necessary for improving their personal and organisational performance, and improving innovation [66]. Therefore, strengthening a culture of learning not only encourages knowledge exchange among employees but also develops their competencies leading to augmenting organisational effectiveness [67].

Recent research has explored how various organisational cultures influence performance through the lens of knowledge sharing, emphasising on attitudes and intentions. Furthermore, the mediating effect of knowledge sharing has been tested, but in different circumstances and with different measurements [68].

This exploration leads to the formulation of the study hypotheses as follows:

**H3a.**  *Organisational culture is positively associated with knowledge-sharing behaviour.*

**H3b.**  *Knowledge-sharing behaviour is positively associated with healthcare workforce performance.*

**H3c.**  *Knowledge-sharing behaviour mediates the association between organisational culture and healthcare workforce performance.*

### 2.4. Organisational Culture, Digital Transformation, and Job Performance

Digital transformation (DT) comprises changing business operations, culture, and organisational elements to meet market demands through digital technologies adoption. This process requires organisations to fundamentally reconsider, reconceptualise, and restructure their entire approach to be suitable for the digital era [69].

Organisational culture should be considered as a key factor, the initial step for a successful digital health transformation process [70]. It encourages organisational members to self-regulate their behaviour based on respect, openness, and trust, thus contributing to the successful implementation of digital health transformation and creating a sustainable competitive advantage [71]. In the context of healthcare, the involvement of healthcare professionals through teamwork ensures that technology aligns with both organisational and employee needs. In addition, it builds a sense of urgency and a proactive attitude towards innovation. This participatory culture also helps organisations anticipate and address the ethical challenges of new technology adoption [72].

Additionally, digital health transformation is a significant mechanism of change. When digital health transformation is integrated with profound shifts in culture and strategy, employees are more likely to embrace these innovations, resulting in holistic performance improvements rather than isolated technological expansions [73]. In other words, whilst technology functions as an enabler, organisations must adapt their structures, processes, capabilities, and culture to create sustainable impact [74], with a strong culture ultimately fostering levels of operational efficiency [75].

Despite the growing importance of digital health transformation [76], empirical evidence regarding its direct effects on individual performance, particularly in public organisations, remains insufficient [77,78]. Most importantly, there is a distinct lack of empirical studies identifying the mediating role of digital health transformation in the relationship between organisational culture-as conceptualised by the Denison model and individual performance in the healthcare sector. Therefore, the following hypotheses are proposed:

**H4a.**  *Organisational culture is positively associated with the adoption of digital health transformation.*

**H4b.**  *Digital transformation is positively associated with healthcare workforce performance.*

**H4c.**  *Digital health transformation mediates the association between organisational culture and healthcare workforce performance.*

## 3. Methods

### 3.1. Research Design, Sampling, and Data Collection

This study adopted a quantitative, non-experimental, cross-sectional survey design, as recommended by Zumitzavan [79], and all constructs were measured concurrently at a single point in time by means of a self-administered questionnaire (Appendix A). The analytical strategy accordingly employs partial least squares structural equation modelling, which is suited to the estimation of complex models containing multiple mediating pathways and higher-order constructs [80,81].

The target population encompassed healthcare professionals—comprising doctors, nurses, medical technicians, pharmacists, administrators, and support staff—within public medical facilities, selected through convenience and snowball sampling methods. Data were collected between June and August 2025 across Ho Chi Minh City and Binh Duong Province, two adjacent localities in southern Vietnam then undergoing administrative consolidation into a single metropolitan health authority. Ho Chi Minh City is the country’s largest urban centre and healthcare hub, with the widest concentration and diversity of public facilities and professional roles, while Binh Duong is a major industrial province whose rapidly expanding, migrant-heavy workforce places distinctive demands on its health system. The two settings thus span the established-metropolitan and high-growth-industrial segments of the region’s public health sector.

The study applied the conventional item-to-respondent ratio of 1:5 [82]; with 68 measurement items, this yields a minimum of 340 respondents. The achieved sample of 450 exceeds the recommended threshold.

Data collection proceeded in three discrete stages involving separate, non-overlapping groups of respondents, comprising 530 participants in total. In the first stage, a pilot test was conducted with 30 health professionals, following the guidance of Hill [83] and Zumitzavan [79], which informed refinements to item wording for linguistic clarity, cultural appropriateness, and alignment with the specific characteristics of the healthcare setting. In the second stage, the revised instrument was administered to a separate group of 50 respondents to confirm preliminary reliability and convergent validity. In the third stage, full-scale data collection was undertaken with a further 450 respondents, which was used for all inferential results reported.

In the third stage, the final questionnaire was administered both in person and via Google Forms, yielding 450 valid responses. Recruitment at this stage followed a defined referral protocol. Five initial contacts were identified from among the research team’s professional contacts working within public healthcare facilities in the two study localities. Individual facilities are not named in this report. The conditions of ethical approval require that participating organisations remain unidentified, the characteristics of the participants are instead described at the level of the four administrative tiers of the Vietnamese public health system, as reported in Table 1. Submissions that were opened but left incomplete were excluded during data screening, and the 450 responses reported represent complete, valid submissions only, for the same reason, a conventional response rate is not reported in this study.

Data were analysed implementing SmartPLS 3.0 with Partial Least Squares Structural Equation Modelling (PLS-SEM) to test variable relationships and effects.

### 3.2. Measurements

Each measurement employed a 6-point Likert scale, ranging from ‘strongly disagree’ (1) to ‘strongly agree’ (6) [84] as adapted from previous research and written in Vietnamese. To ensure the equivalence of the Vietnamese version compared to the original English version, the study uses the back-translation method [85].

This study utilised Fey & Denison [86]’s thirty-six-item scale to assess the four dimensions of OCU including Involvement, Consistency, Adaptability, and Mission. Jang et al. [56]’s four-item scale was used to measure the characteristics of OC. KSB was measured by five-item scale from Blouch et al. [87] and Bock et al. [88]. DT was assessed by five-item scale from Nasiri et al. [69] and AlNuaimi et al. [89]. JP was evaluated by employees through eighteen-item scale from Shahidi et al. [44] with two aspects of In-Role Performance and Organisational Citizenship Behaviours (Appendix B).

## 4. Results

### 4.1. Measurement Model Assessment

#### 4.1.1. Evaluation of First-Order Constructs in the Outer Model

**Figure 1:**
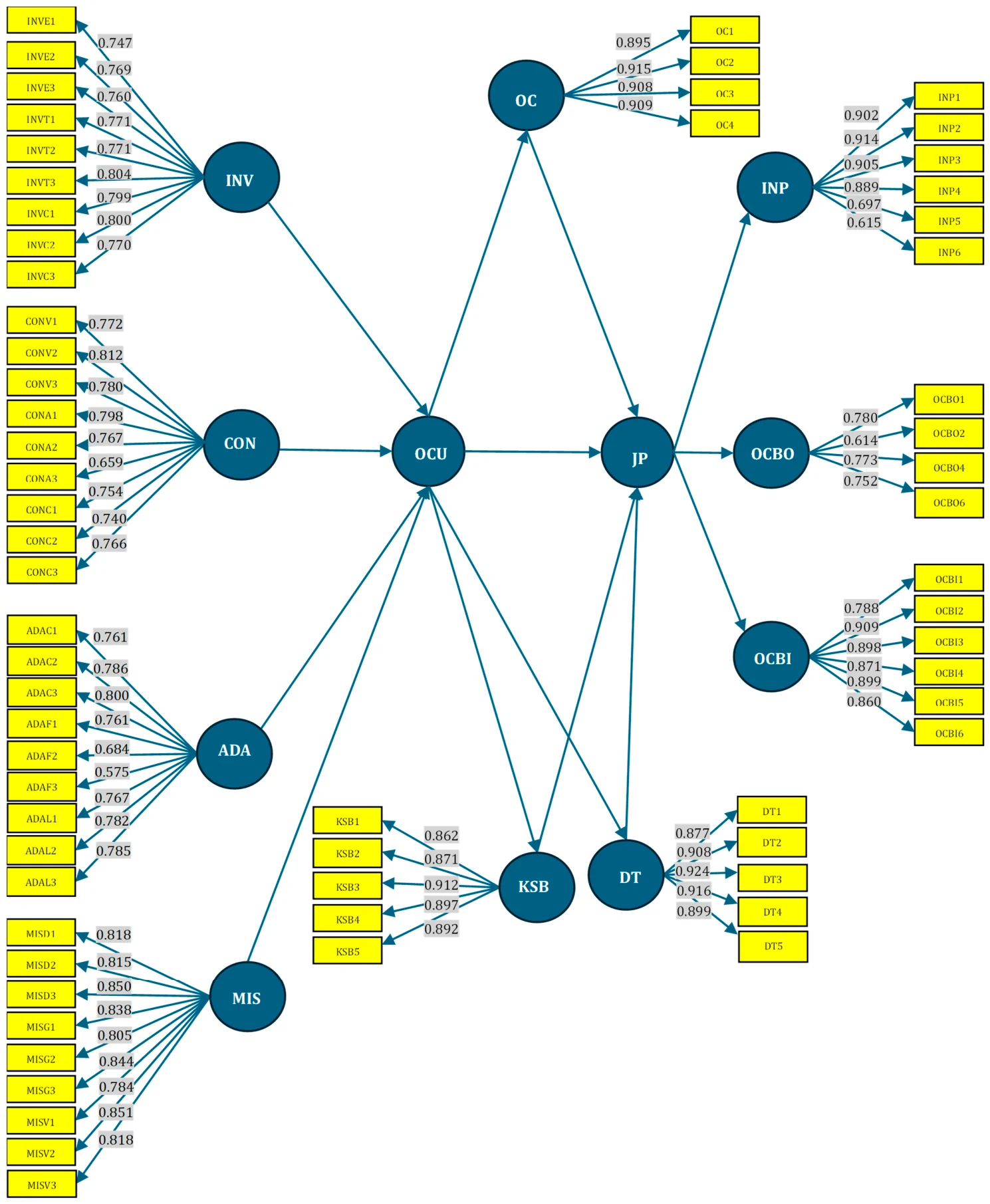
Measurement model.

Three measures were used to assess the measurement model. Outer loading shows how strongly an observed variable relates to its latent variable. Composite Reliability (CR) measures internal consistency reliability, and Average Variance Extracted (AVE) measures convergent validity [81]. As shown in Table 2, most of observed variables had outer loadings above 0.7, indicating good indicator reliability. A few loaded lower, between 0.4 and 0.708, including ADAF2, ADAF3, CONA3, INP5, INP6, and some OCBO items. For indicators in this range, Hair et al. [81] advise keeping them unless removing them would raise the construct’s CR or AVE above the required thresholds and removing them only when the constructs fail those thresholds. We followed this rule for all such indicators. Under this rule, ADAF2, ADAF3, CONA3, INP5, and INP6 were kept, because their constructs still met the AVE threshold despite the lower loadings (ADA = 0.559, CON = 0.581, INP = 0.687). OCBO was the only construct that fell short, with an initial AVE of 0.410 below the 0.50 threshold. Its two weakest items, OCBO3 and OCBO5, were removed, which raised the construct’s AVE to 0.537 and CR to 0.821. As reported in Table 2, the retained OCBO items then loaded at 0.780 (OCBO1), 0.773 (OCBO4), 0.752 (OCBO6), and 0.614 (OCBO2); OCBO2 was kept because the construct already met the reliability and validity thresholds, so no further items were removed. After this step, the CR and AVE of every latent variable were above the 0.7 and 0.5 thresholds, and all retained indicators were kept in the research model [81] (Figure 1).

The digital transformation construct returned a composite reliability of 0.958, slightly above the 0.95 upper bound recommended by Hair et al. [81], which can signal redundancy among items. However, its AVE of 0.819 confirms convergent validity, and its VIF values (3.133 to 4.780) stayed below the threshold of 5. The construct was kept in its original form for two reasons. First, the scale was used unchanged from validated instruments [69,89] and shortening it would reduce comparability with prior research. Second, its five items measure closely related aspects of the same transformation process, so some overlap is expected rather than a sign of poor scale design. The high reliability is therefore reported as a feature of the instrument, not a flaw in the measurement model.

Discriminant validity shows how distinct each construct is from the others and was assessed using the Heterotrait-Monotrait Ratio (HTMT) [81]. As shown in Table 3, all HTMT values were below 0.90, the threshold recommended by Henseler et al. [90], so discriminant validity was established.

Four ratios among the Denison dimensions were higher, at or above the stricter 0.85 threshold: ADA-MIS (0.893), CON-ADA (0.891), CON-MIS (0.842), and INV-ADA (0.836). Henseler et al. [90] apply the 0.85 threshold to constructs that are conceptually distinct and the 0.90 threshold to those that are conceptually similar. The Denison dimensions are related facets of a single cultural system, so the 0.90 threshold is the appropriate benchmark. For the same reason, organisational culture is modelled as a higher-order construct that combines the four dimensions, and they need not be fully distinct from one another. As a further check on these borderline pairs, a bootstrap procedure was run: the 95% confidence intervals for the HTMT values did not include 1, confirming that no two constructs are identical.

#### 4.1.2. Evaluation of Second-Order Constructs in the Outer Model

Job performance is modelled as a second-order reflective construct comprising in-role performance and two forms of organisational citizenship behaviour. This follows the established conceptualisation of job performance as two principal dimensions, task performance and contextual performance, the latter commonly operationalised as organisational citizenship behaviour [42,91]. Citizenship behaviour is thus a constituent domain of job performance rather than an adjunct to it. The second-order specification rests on the premise that the three dimensions covary because they share a common source, namely the employee’s overall contribution to organisational effectiveness. Their conceptual distinctiveness is retained in the model: in-role performance, organisation-directed and individual-directed citizenship behaviour are each estimated as separate first-order factors rather than pooled into a single scale, so the higher-order construct summarises their shared variance without treating the dimensions as equivalent. The boundary between task and citizenship performance is also less sharp in practice than in principle, since conscientious initiative is at once task-directed and discretionary.

The empirical results support this specification. For the JP construct, all three dimensions loaded above 0.7 (INP = 0.912, OCBO = 0.794, OCBI = 0.869), and the construct met the thresholds for reliability (CR = 0.894) and convergent validity (AVE = 0.739) (Table 2). The AVE of 0.739 means the second-order construct explains about 74% of the variance in its three dimensions, consistent with their acting as facets of one higher-order construct. Organisation-directed and individual-directed citizenship behaviour were kept as two separate dimensions rather than merged into one, which preserves the distinction between the two targets of discretionary behaviour; their similar loadings (OCBO = 0.794, OCBI = 0.869) support this choice. The three dimensions also remain empirically distinguishable from one another: their HTMT rations were 0.776 (INP-OCBO), 0.747 (INP-OCBI) and 0.582 (OCBO-OCBI), all below the conservative 0.85 criterion (Table 3). The dimensions are therefore sufficiently related to be summarised by a common higher-order construct, yet sufficiently distinct to warrant retention as separate first-order factors.

For the formative construct, the measurement model was assessed through outer weights and indicator collinearity. Significant outer weights show that the indicators meaningfully contribute to the construct [81]. At the 5% level, all four indicators (ADA, CON, INV and MIS) had significant outer weights (*p*-value < 0.05), confirming their contribution to organisational culture (OCU). Their VIF values ranged from 2.944 to 4.349, all below the recommended threshold of 5, which indicates no critical multicollinearity and confirms that each indicator adds distinct explanatory information [81,92].

#### 4.1.3. Assessment of Common Method Bias

Because all constructs were measured through a single self-administered instrument completed by one respondent at one point in time, the potential for common method bias was assessed both procedurally and statistically. Procedural remedies were built into the design. Participation was voluntary and responses were anonymous, with no identifier linking a response to an individual, facility or referral source. This reduces the incentive to give socially desirable answers on items about respondents’ own organisations and supervisors. The instrument was also assembled from previously validated scales and translated using back-translation [85], which limits ambiguous item wording as a source of systematic response bias.

Two statistical assessments were then conducted. First, Harman’s single-factor test was performed by subjecting all 66 retained indicators to an unrotated principal components analysis with extraction constrained to a single factor. The first component accounted for 46.38% of the total variance, below the conventional 50% criterion, indicating that no single factor dominates the covariance among the measures. Second, collinearity among the structural model constructs was examined as a supplementary indication, since severe collinearity may arise where responses reflect a common source rather than distinct constructs [93]. The inner VIF values were 2.209 (OC), 2.413 (KSB), 2.409 (DT), and 3.523 (OCU), all below the threshold of 5 recommended by Hair et al. [81], and three of the four also fall below the more conservative criterion of 3.3 (Table 4). Each construct therefore retains distinct explanatory information within the structural model. Taken together with the Harman’s test and the procedural safeguards described above, these results indicate that common method bias is unlikely to threaten the validity of the estimates, although it cannot be excluded entirely given the single-source design.

### 4.2. Structural Model Assessment

Collinearity Assessment. All inner VIF values were below the recommended threshold of 5 (Table 4), indicating the absence of critical multicollinearity and confirming that each predictor contributed distinct explanatory information to the model [81].

Coefficient of Determination (R^2^). The model suggested moderate-to-substantial explanatory power. OCU explained 50.4%, 54.7%, and 56.5% of the variance in OC, KSB, and DT, respectively, whilst the full model accounted for 66.3% of the variance in JP, indicating strong explanatory capability [81].

Predictive Relevance (Q^2^). The Q^2^ values for OC, KSB, DT, and JP ranged from 0.409 to 0.476, confirming satisfactory predictive relevance [80]. Furthermore, the PLSpredict analysis indicated that most indicators exhibited lower RMSE and MAE values than the linear benchmark model (Table 5), signifying moderate out-of-sample predictive power and meaningful predictive capability of the structural model [81,94].

Effect Sizes (f^2^). The effects of OCU on OC, KSB, and DT were substantial (f^2^ values ranging from 1.015 to 1.297), highlighting its dominant explanatory role. Regarding JP, KSB exerted the strongest effect (f^2^ = 0.230), whereas OCU and DT demonstrated smaller effects and OC showed a negligible contribution [95,96]. Collectively, these findings underscore the pivotal role of KSB in reinforcing the relationship between OCU and JP (Table 6).

### 4.3. Path Coefficients and Hypotheses Testing

**Figure 2:**
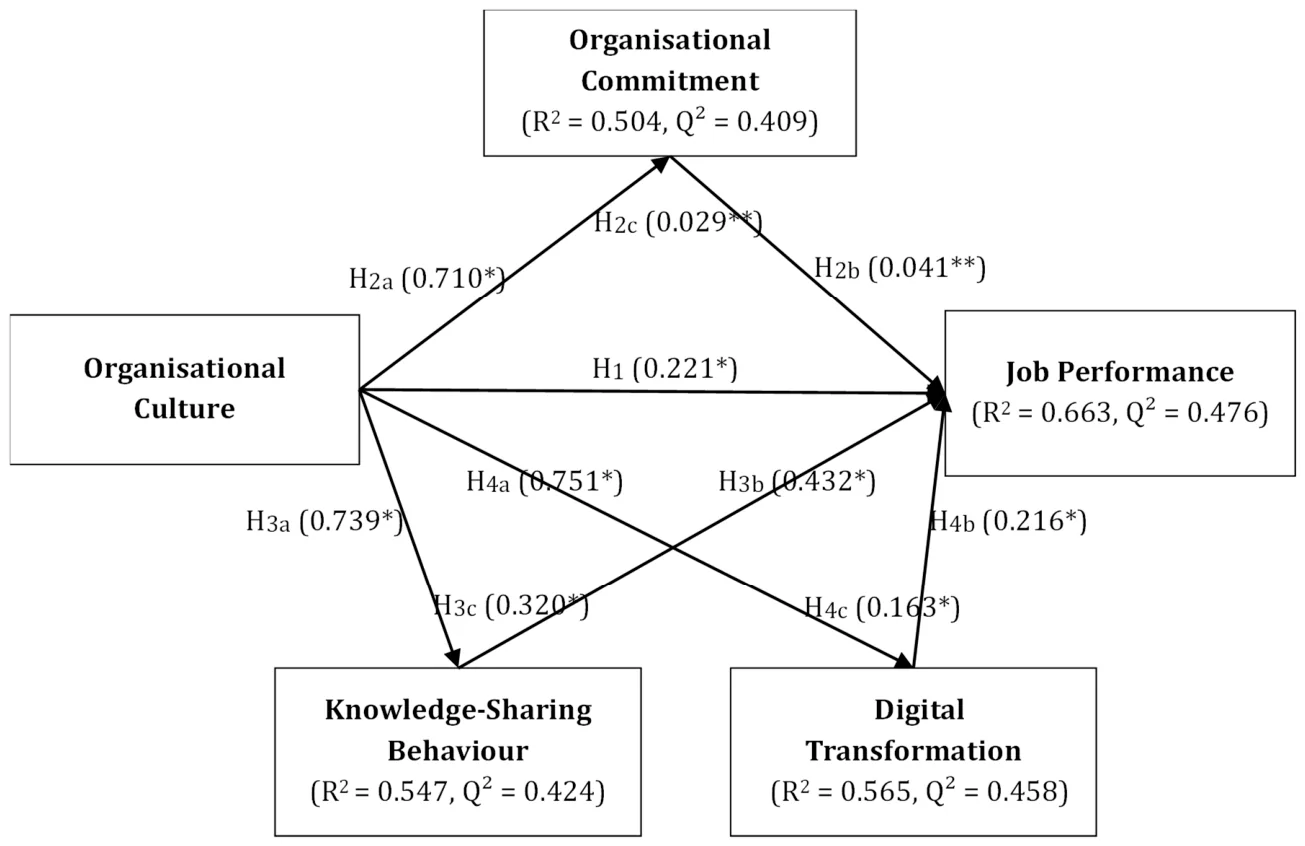
Structural model. * *p*-value < 0.05, ** *p*-value > 0.05.

The bootstrapping technique was used to assess the path coefficients’ significance, with 5000 replications of subsamples at the 95% confidence interval. Table 6 indicates that the path coefficients in the model are statistically significant, except for the relationship between OC and JP (*p*-value > 0.05). Specifically, the path from OCU → JP shows β = 0.221, *p*-value < 0.05, suggesting that OCU has a direct positive effect on JP, thus supporting Hypothesis H1. The path from OCU → OC reveals β = 0.710, *p*-value < 0.05, confirming the positive and significant effect of OCU on OC and supporting Hypothesis H2a. Likewise, hypotheses H3a (OCU → KSB, β = 0.739, *p*-value < 0.05), H4a (OCU → DT, β = 0.751, *p*-value < 0.05), H3b (KSB → JP, β = 0.432, *p*-value < 0.05), H4b (DT → JP, β = 0.216, *p*-value < 0.05) are all supported, and the effects are positive [81] (Figure 2). Thus, examining the impact on JP reveals that the order of impact from strong to weak on JP is KSB (β = 0.432) > OCU (β = 0.221) > DT (β = 0.216). In other words, the KSB has the most significant and most powerful on JP, followed by the impact of OCU on JP, whilst the impact of DT on JP is only weak.

### 4.4. Mediation Analysis

The mediating mechanisms from OCU to JP through OC is not supported (β = 0.029, *p*-value > 0.05). Although OCU was significantly associated with OC, the absence of a significant OC-JP association means that no indirect association is transmitted through this pathway. By contrast, the mechanisms through KSB and DT were both statistically significant (*p*-value < 0.05). The pathway through KSB was the stronger of the two (β = 0.320), whilst the pathway through DT was weaker (β = 0.163) (Table 6). A significant direct association also remained between OCU and JP (β = 0.221). These conclusions are corroborated by bias-corrected bootstrap confidence intervals obtained from 5000 subsamples at the 95% level. The interval for the pathway through OC contained zero (−0.044 to 0.103), confirming that no significant indirect association operates through this route, whereas the intervals for the pathways through KSB (0.234 to 0.413) and DT (0.067 to 0.263) both excluded zero, confirming the significance of these indirect associations. Since the direct association between OCU and JP remains significant alongside both indirect associations, and all three operate in the same direction, KSB and DT exhibit complementary partial mediation, as proposed by Demming et al. [97].

In conclusion, the total effect of OCU on JP is 0.733. Through bootstrapping, this effect is statistically significant (*p*-value < 0.05). This effect is mainly made up of the direct effect (β = 0.221) and the indirect effects through KSB (β = 0.320) and DT (β = 0.163). In addition, the model also shows an indirect effect through OC, however, this indirect effect is not strong enough to reach statistical significance in the study sample (β = 0.029, *p*-value > 0.05).

## 5. Discussion

This section interprets the findings in relation to the research question, considers their theoretical and practical significance, and acknowledges the limitations of the study. Section 5.1 discusses the direct and indirect associations identified in the structural model, Section 5.2, Section 5.3 and Section 5.4 set out the contributions and implications, and Section 5.5 outlines limitations and directions for future research.

### 5.1. Interpretation of Findings

Improved healthcare workforce performance may ultimately contribute to stronger patient-care quality, service coordination, healthcare responsiveness, and operational effectiveness within public healthcare organisations.

Organisational culture is substantially associated with the performance of healthcare professionals, consistent with prior studies [84,98,99]. In healthcare surroundings, a culture grounded in shared values, consistency and ethical discipline helps minimise medical errors whilst bolstering operational reliability. Simultaneously, empowerment, leadership support and collaborative teamwork fortify professional autonomy and psychological safety, enabling healthcare professionals to prioritise patient care excellence. Moreover, a learning-oriented and mission-driven culture enhances organisational resilience and adaptability, eventually sustaining high workforce performance.

Organisational culture is significantly associated with organisational commitment, in a pattern consistent with Social Exchange Theory and prior evidence across multiple sectors [100,101], particularly within the health contexts [102]. Given the inherently meaningful nature of healthcare work, a strong organisational culture nurtures trust, shared values and collective purpose, thereby reinforcing employees’ long-term commitment and dedication to the organisation.

Interestingly, neither the direct association between organizational commitment and healthcare workforce performance nor the indirect association transmitted through commitment reached statistical significance, contrary to public-sector evidence [61]. However, these findings are consistent with prior research, supported by thoughtful arguments. For instance, Bektiarso [103], who argued that performance is a formal job responsibility that must be completed regardless of employee commitment. Diamantidis and Chatzoglou [49] also demonstrated that commitment is unrelated to employee performance due to the presence of moderating variables. Based on Public Service Motivation Theory [104], employee in mission-driven organisations such as hospitals are motivated primarily by a desire to serve patient and their profession, rather than by attachment to a specific employer. Professional ethics and clinical protocols and regulatory obligations standardise their effort independently of organisational attachment. Furthermore, Situational Constraints Theory suggests that when the work environment limits what an employee can accomplish, performance is bounded by those constraints rather than by a willingness to exert effort [105]. Vietnamese healthcare industry operates under workforce shortages and infrastructural limitations [106]. During healthcare delivery, medical staff face severe job stress and a prevalent burnout due to persistent demand-resource imbalances [107]. Therefore, a highly committed professional and a less committed colleague may produce similar outputs because both operate at the performance ceiling imposed by available time, staffing, and equipment. Commitment thus appears to operate as an outcome of a supportive culture and a contributor to retention rather than as the direct driver to performance.

On the other hand, knowledge-sharing behaviour significantly mediated the nexus between organisational culture and healthcare workforce performance. Consistent with SET and TPB, a supportive organisational culture characterised by strategic leadership, organisational learning, collaboration and innovation heartens healthcare professionals to energetically exchange knowledge and improve professional expertise. Given the relatively young and development-oriented workforce in this study, knowledge sharing appears particularly critical for reinforcing adaptability, continuous learning and patient care quality.

Digital transformation was identified as a significant mediator between organisational culture and healthcare workforce performance. A strong organisational culture facilitates digital transformation by aligning digital initiatives with strategic objectives, fostering employee engagement and heartening adaptability towards emerging technologies. Consistent with prior research [108], digital transformation subsequently enhances workforce performance by streamlining workflows, improving data quality and accelerating decision-making processes. This finding also addresses the research gap suggested by Ly [77] regarding the direct performance implications of digital transformation. By mitigating manual tasks and improving professional autonomy, digital technologies allow healthcare professionals to operate more efficiently, contribute to organisational development and strengthen collaborative support through digital platforms. In lower and middle-income settings, digital infrastructure, interoperability and clinical information systems are still maturing [109]. The performance returns to digital adoption may therefore be constrained by the underlying technical environment rather than by workforce willingness [110]. By contrast, knowledge sharing is deeply rooted in interpersonal communication and organisational socialisation. Therefore, it can often be enacted through existing professional and team routines, requiring comparatively less reliance on robust technical infrastructure. This may help explain why knowledge-sharing behaviour transmitted a substantially larger share of the association between culture and performance than digital health transformation did in the present sample.

### 5.2. Contributions of Research Findings

This study integrates organisational, psychological, behavioural and technological dimensions to enlighten healthcare workforce performance at the individual level. In doing so, it strengthens organisational behaviour and healthcare workforce management theories whilst validating the on-going relevance of these theoretical perspectives across contemporary healthcare contexts.

Additionally, the study offers novel empirical evidence regarding the effects of organisational culture dimensions—mission, involvement, consistency and adaptability—on healthcare workforce performance through the mediating roles of knowledge-sharing behaviour and digital health transformation. The varying strengths of these relationships provide valuable strategic insights for public administrators pursuing to improve workforce performance within the Vietnamese healthcare sector.

### 5.3. Theoretical Implications

Previous studies have primarily focused on assessing organisational culture or examining the effects of culture types on organisational performance. In contrast, this study advances a more employee-centred perspective by discovering how dimensions of a strong organisational culture improve healthcare workforce performance. This underscores the importance of employees’ perceptions as a critical feedback mechanism for organisational culture development and strategic adjustment.

Moreover, the integration of knowledge-sharing behaviour and digital health transformation extends the existing literature by presenting contextually relevant mediating mechanisms within the Vietnamese healthcare sector. The non-significant mediating role of organisational commitment further recommends that additional psychological, behavioural or contextual mechanisms may shape the culture–performance relationship, thereby offering valuable directions for future research.

Finally, the application of Social Exchange Theory and the Theory of Planned Behaviour broadens the theoretical understanding of the anticipated relationships and supports the extension of these frameworks to other sectors and organisational contexts characterised by differing leadership styles, institutional environments and regional cultures.

### 5.4. Practical Implications

The findings recommend that healthcare organisations pursuing to improve digital transformation should prioritise the development of a strong organisational culture characterised by a clear mission, consistency, employee involvement, and adaptability. A well-articulated strategic direction lessens ambiguity, aligns organisational members around common objectives, and facilitates effective strategy execution. Accordingly, leaders should embed organisational missions within formal systems and operational processes whilst ensuring that strategic priorities are translated into measurable objectives. Regular communication through town halls, digital dashboards, and internal communication channels can further reinforce strategic alignment and employee responsibility.

The results also emphasise the importance of organisational consistency and trust as foundations for knowledge sharing and digital transformation. Senior leaders should establish behavioural congruence by aligning organisational values with strategic decisions, human resource practices, and day-to-day operations. Such alignment helps nurture the trust on which organisational learning and collaborative knowledge sharing depend. Recognition and reward systems should also acknowledge contributions to knowledge creation, cross-functional collaboration, and digital innovation. Rewarding these contributions reduces knowledge hoarding and strengthening employee engagement in transformation initiatives.

Given the rapidly evolving healthcare environment, organisations should continuously strengthen their adaptive capabilities and digital readiness. Regular reviews of organisational structures, processes, and risk management systems can enhance responsiveness to technological developments, changing patient expectations, and emerging healthcare challenges. A culture that embraces adaptability and continuous learning is likely to accelerate digital transformation efforts by encouraging organisational agility, data-driven decision-making, and employee responsiveness to change. On the other hand, resistance to change and weak cultural alignment may undermine transformation initiatives and limit long-term organisational effectiveness.

Lastly, healthcare organisations should invest strategically in knowledge management infrastructure and digital capability development. Training programmes focused on digital literacy, data governance, and emerging technologies can reinforce workforce readiness and support the successful adoption of digital transformation initiatives. Among the pathway examined in this study, knowledge-sharing behaviour was the strongest contributor to workforce performance, carrying the largest share of the total effect of organisational culture (β = 0.320) and the largest effect size (f^2^ = 0.230) (Table 6). Where resources are constrained, healthcare organisations should therefore prioritise investment in collaborative and knowledge-exchange practices, pursuing digital initiatives alongside rather than in place of knowledge-sharing measures. In addition, the establishment of centralised knowledge repositories, supported by dedicated knowledge management personnel, can facilitate the effective capture and dissemination of both tacit and explicit knowledge [111]. The integration of artificial intelligence-enabled tools, comprising intelligent assistants and conversational chatbots, may further improve knowledge accessibility, improve decision quality, and support the delivery of high-quality patient care [112].

### 5.5. Limitations and Recommendations for Future Research

Several limitations should be acknowledged. First, the use of a cross-sectional design with self-reported data collected at a single point in time means that common method variance cannot be entirely ruled out, and causal relationships cannot be definitively established. Second, the structural model did not include control variables, suggesting that the observed relationships might be partially influenced by demographic and organisational characteristics. Importantly, the null result regarding the relationship between organisational commitment and performance should be interpreted with statistical power in mind; this finding may simply indicate the absence of a practically meaningful effect rather than confirming that no relationship exists at all [113]. Finally, because the study utilises convenience and snowball sampling in public facilities in two Vietnamese cities, nursing staff were over-represented. Since nursing work involves high interdependence and continuous patient contact, the prominence of the knowledge-sharing pathway may partly reflect this composition, and the findings are most directly applicable to nursing and allied clinical staff in public facilities.

These limitations highlight valuable directions for future research. Subsequent studies would benefit from employing longitudinal or experimental designs, combined with stratified sampling and larger sample sizes, to strengthen causal claims and external validity. Future research should also conduct multi-group analyses to explore how these structural relationships might differ across various occupational groups and healthcare facility types (e.g., public, private, and military). Finally, future models might also incorporate further individual and job-related factors, such as competencies and person–job fit, and additional mediators including employee engagement, well-being and innovation, with the non-significant role of organisational commitment warranting particular attention in relation to job stress and burnout.

## 6. Conclusions

The psychological and behavioural determinants of health workforce performance remain a compelling area of enquiry, particularly as Vietnam’s public health sector undergoes rapid digitalisation and rising service demand. Using a quantitative, cross-sectional design, this study found that organisational culture is associated with healthcare workforce performance both directly and indirectly. Knowledge-sharing behaviour and digital health transformation were identified as significant mediating mechanisms in this context, whereas organisational commitment was not. Interestingly, these factors exert different levels of impact on job performance of healthcare professionals. This study also provides managerial implications in both academic and practical aspects as a minor contribution to the research on organisational behaviour, especially in the healthcare sector of Vietnam.

## Figures and Tables

**Table 1:** Demographic Characteristics of the Respondents.

**Demographic Variables**	**Categories**	**Frequency** (Participants)	**Percentage (%)**
Gender	Male	117	26.0
	Female	333	74.0
Age group (years old)	<25	32	7.1
	25–30	96	21.3
	Greater than 30–35	91	20.2
	Greater than 35–40	97	21.6
	Greater than 40–45	69	15.3
	Greater than 45–50	26	5.8
	Greater than 50–55	21	4.7
	Greater than 55–60	12	2.7
	>60	6	1.3
Education level	College	160	35.6
	Bachelor	249	55.3
	Master’s	35	7.8
	Doctorate	6	1.3
Job position	Clinician	85	18.9
	Nurse	289	64.2
	Medical technician	21	4.7
	Pharmacist	10	2.2
	Management	20	4.4
	Non-clinician staff	25	5.6
Tenure (years)	<3	55	12.2
	3–5	72	16.0
	Greater than 5–10	86	19.1
	Greater than 10–20	163	36.2
	>20	74	16.4
Health facility level	Central level	75	16.7
	Level of provinces and centrally run cities	255	56.7
	Level of districts, towns and provincial cities	99	22.0
	Level of communes, wards and townships	21	4.7
Work location	Ho Chi Minh City	254	56.4
	Binh Duong Province	196	43.6

**Table 2:** Indicator Reliability, Internal Consistency Reliability, and Convergent Validity.

Construct	Item	Outer Loading	Composite Reliability (CR)	Average Variance Extracted (AVE)
First-order reflective constructs				
Job Performance (JP)				
In-role Performance (INP)	INP1	0.902	0.928	0.687
	INP2	0.914		
	INP3	0.905		
	INP4	0.889		
	INP5	0.697		
	INP6	0.615		
Organisational Citizenship Behaviours toward Organisation (OCBO)	OCBO1	0.780	0.821	0.537
	OCBO2	0.614		
	OCBO4	0.773		
	OCBO6	0.752		
Organisational Citizenship Behaviours toward Individuals (OCBI)	OCBI1	0.788	0.950	0.760
	OCBI2	0.909		
	OCBI3	0.898		
	OCBI4	0.871		
	OCBI5	0.899		
	OCBI6	0.860		
Organisational Culture (OCU)				
Involvement (INV)	INVE1	0.747	0.932	0.604
	INVE2	0.769		
	INVE3	0.760		
	INVT1	0.771		
	INVT2	0.771		
	INVT3	0.804		
	INVC1	0.799		
	INVC2	0.800		
	INVC3	0.770		
Consistency (CON)	CONV1	0.772	0.925	0.581
	CONV2	0.812		
	CONV3	0.780		
	CONA1	0.798		
	CONA2	0.767		
	CONA3	0.659		
	CONC1	0.754		
	CONC2	0.740		
	CONC3	0.766		
Adaptability (ADA)	ADAC1	0.761	0.919	0.559
	ADAC2	0.786		
	ADAC3	0.800		
	ADAF1	0.761		
	ADAF2	0.684		
	ADAF3	0.575		
	ADAL1	0.767		
	ADAL2	0.782		
	ADAL3	0.785		
Mission (MIS)	MISD1	0.818	0.950	0.681
	MISD2	0.815		
	MISD3	0.850		
	MISG1	0.838		
	MISG2	0.805		
	MISG3	0.844		
	MISV1	0.784		
	MISV2	0.851		
	MISV3	0.818		
Organisational Commitment (OC)	OC1	0.895	0.949	0.823
	OC2	0.915		
	OC3	0.908		
	OC4	0.909		
Knowledge-Sharing Behaviour (KSB)	KSB1	0.862	0.949	0.787
	KSB2	0.871		
	KSB3	0.912		
	KSB4	0.897		
	KSB5	0.892		
Digital Transformation (DT)	DT1	0.877	0.958	0.819
	DT2	0.908		
	DT3	0.924		
	DT4	0.916		
	DT5	0.899		
Second-order reflective construct				
Job Performance (JP)	INP	0.912	0.894	0.739
	OCBO	0.794		
	OCBI	0.869		

**Table 3:** Appraising discriminant validity through HTMT.

	INP	OCBO	OCBI	INV	CON	ADA	MIS	OC	KSB	DT
INP										
OCBO	0.776									
OCBI	0.747	0.582								
INV	0.657	0.579	0.685							
CON	0.617	0.564	0.651	0.821						
ADA	0.718	0.593	0.733	0.836	0.891					
MIS	0.629	0.534	0.627	0.787	0.842	0.893				
OC	0.612	0.560	0.582	0.675	0.699	0.718	0.717			
KSB	0.782	0.652	0.713	0.727	0.709	0.748	0.718	0.700		
DT	0.636	0.551	0.720	0.695	0.702	0.770	0.759	0.656	0.670	

**Table 4:** Inner VIF Values.

	OC	KSB	DT	JP
OCU	1.000	1.000	1.000	3.523
OC				2.209
KSB				2.413
DT				2.409

**Table 5:** PLS Predict.

	PLS-RMSE	LM-RMSE	PLS-MAE	LM-MAE	MAPE	Q^2^_Predict
OC1	0.664	0.668	0.427	0.429	11.780	0.387
OC2	0.705	0.710	0.486	0.488	13.053	0.394
OC3	0.590	0.591	0.412	0.414	9.459	0.378
OC4	0.595	0.594	0.414	0.409	9.856	0.449
KSB1	0.601	0.597	0.418	0.422	9.333	0.332
KSB2	0.544	0.548	0.386	0.390	8.449	0.394
KSB3	0.491	0.494	0.364	0.362	7.750	0.486
KSB4	0.498	0.498	0.374	0.370	7.874	0.485
KSB5	0.507	0.509	0.376	0.374	7.750	0.406
DT1	0.535	0.537	0.389	0.391	8.517	0.423
DT2	0.524	0.525	0.382	0.383	8.420	0.464
DT3	0.537	0.540	0.383	0.387	8.708	0.470
DT4	0.550	0.552	0.394	0.396	8.669	0.470
DT5	0.564	0.568	0.414	0.417	8.866	0.452
INP	0.753	0.753	0.582	0.577	175.546	0.436
OCBO	0.865	0.866	0.670	0.673	115.131	0.256
OCBI	0.732	0.727	0.542	0.533	138.456	0.465

**Table 6:** Hypotheses Testing Results.

Hypothesis	Path	Βeta (β)	t-Values	*p*-Values	f^2^/95% BCa CI	Decision	Effect Size
Total effect							
	OCU -> JP	0.733	24.764	0.000			
Direct effects							
H1	OCU -> JP	0.221	3.190	0.001	0.041	Supported	Small
H2a	OCU -> OC	0.710	19.600	0.000	1.015	Supported	Large
H3a	OCU -> KSB	0.739	28.607	0.000	1.206	Supported	Large
H4a	OCU -> DT	0.751	23.099	0.000	1.297	Supported	Large
H2b	OC -> JP	0.041	0.794	0.427	0.002	Rejected	No impact
H3b	KSB -> JP	0.432	7.227	0.000	0.230	Supported	Medium
H4b	DT -> JP	0.216	3.271	0.001	0.058	Supported	Small
Indirect effects							
H2c	OCU -> OC -> JP	0.029	0.783	0.433	[−0.044, 0.103]	Rejected	
H3c	OCU -> KSB -> JP	0.320	7.080	0.000	[0.234, 0.413]	Supported	
H4c	OCU -> DT -> JP	0.163	3.234	0.001	[0.067, 0.263]	Supported	

Note: For direct effects, the sixth column reports the f^2^ effect size; for indirect effects, it reports the 95% bias-corrected and accelerated bootstrap confidence interval (5000 subsamples). An interval excluding zero indicates a statistically significant indirect association.

## Data Availability

The data presented in this study are available on request from the corresponding author. The data are not publicly available because they contain information that could compromise the privacy of individual healthcare professionals who participated in the survey.

## Appendix A. STROBE Statement—Checklist for Cross-Sectional Studies

Manuscript title: Organisational Culture and Healthcare Workforce Performance in Vietnam: The Mediating Roles of Knowledge Sharing and Digital Transformation

Study design: Quantitative, non-experimental, cross-sectional survey

**Table A1:** STROBE checklist for cross-sectional studies.

Section	Item No.	Recommendation	Reported in Manuscript
Title and abstract	1	(a) Indicate the study’s design with a commonly used term in the title or the abstract	Title, Abstract—cross-sectional design
		(b) Provide in the abstract an informative and balanced summary of what was done and what was found	Abstract
Introduction			
Background/rationale	2	Explain the scientific background and rationale for the investigation being reported	Section 1
Objectives	3	State specific objectives, including any prespecified hypotheses	Section 1; Section 2—hypotheses H1–H4c
Methods			
Study design	4	Present key elements of study design early in the paper	Section 3.1—quantitative, non-experimental, cross-sectional design
Setting	5	Describe the setting, locations, and relevant dates, including periods of recruitment, exposure, follow-up, and data collection	Section 3.1—public healthcare facilities in Ho Chi Minh City and Binh Duong Province, southern Vietnam; data collected June–August 2025
Participants	6	(a) Give the eligibility criteria, and the sources and methods of selection of participants	Section 3.1—healthcare professionals in public medical facilities; convenience and snowball sampling with five defined seeds
Variables	7	Clearly define all outcomes, exposures, predictors, potential confounders, and effect modifiers. Give diagnostic criteria, if applicable	Section 2; Section 3.2; Figure 1. Predictor: OCU. Mediators: OC, KSB, DT. Outcome: JP
Data sources/ measurement	8	For each variable of interest, give sources of data and details of methods of assessment (measurement). Describe comparability of assessment methods if there is more than one group	Section 3.2—validated multi-item scales; six-point Likert format; back-translation [85]
Bias	9	Describe any efforts to address potential sources of bias	Section 3.1 (anonymity, voluntary participation, pilot testing); Section 4.1.3—procedural remedies, Harman single-factor test, structural model collinearity
Study size	10	Explain how the study size was arrived at	Section 3.1—item-to-respondent ratio 1:5 [82]; 68 items yield minimum 340; achieved 450
Quantitative variables	11	Explain how quantitative variables were handled in the analyses. If applicable, describe which groupings were chosen and why	Section 3.2; Section 4.1—latent variables in PLS-SEM; JP as second-order reflective construct; OCU as second-order formative construct
Statistical methods	12	(a) Describe all statistical methods, including those used to control for confounding	Section 3.1; Section 4—PLS-SEM using SmartPLS 3.0
		(b) Describe any methods used to examine subgroups and interactions	Not applicable—no subgroup or interaction analyses; multi-group analysis noted as future research (Section 5.5)
		(c) Explain how missing data were addressed	Section 3.1—incomplete submissions excluded at screening; complete cases analysed
		(d) If applicable, describe analytical methods taking account of sampling strategy	Not applicable—non-probability sampling; no survey weights. Limitation in Section 5.5
		(e) Describe any sensitivity analyses	Section 4.1.1—bootstrap CIs for HTMT; Section 4.2—bias-corrected bootstrapping, 5000 subsamples
Results			
Participants	13	(a) Report numbers of individuals at each stage of study—eg numbers potentially eligible, examined for eligibility, confirmed eligible, included in the study, completing follow-up, and analysed	Section 3.1—pilot (*n* = 30), preliminary validation (*n* = 50), main survey (*n* = 450); total 530
		(b) Give reasons for non-participation at each stage	Section 3.1—incomplete submissions excluded; response rate not reported as referral protocol yields no fixed denominator
		(c) Consider use of a flow diagram	Not included; three-stage recruitment described in Section 3.1
Descriptive data	14	(a) Give characteristics of study participants (eg demographic, clinical, social) and information on exposures and potential confounders	Table 1—demographic and organisational characteristics of the 450 respondents
		(b) Indicate number of participants with missing data for each variable of interest	Section 3.1—complete cases; no item-level missing data
Outcome data	15	Report numbers of outcome events or summary measures	Section 4.1—measurement model results (Table 2 and Table 3)
Main results	16	(a) Give unadjusted estimates and, if applicable, confounder-adjusted estimates and their precision (e.g., 95% confidence interval). Make clear which confounders were adjusted for and why they were included	Section 4.2—path coefficients, significance and bootstrap CIs (Table 4, Table 5 and Table 6); no control variables entered (see Section 5.5)
		(b) Report category boundaries when continuous variables were categorized	Not applicable
		(c) If relevant, consider translating estimates of relative risk into absolute risk for a meaningful time period	Not applicable—no risk estimates
Other analyses	17	Report other analyses done—eg analyses of subgroups and interactions, and sensitivity analyses	Section 4.2—mediation analysis of indirect effects through OC, KSB and DT; total effects
Discussion			
Key results	18	Summarise key results with reference to study objectives	Section 5.1; Section 6
Limitations	19	Discuss limitations of the study, taking into account sources of potential bias or imprecision. Discuss both direction and magnitude of any potential bias	Section 5.5—common method variance; cross-sectional design; absence of control variables; non-probability sampling; single-region scope, statistical power
Interpretation	20	Give a cautious overall interpretation of results considering objectives, limitations, multiplicity of analyses, results from similar studies, and other relevant evidence	Section 5.1, Section 5.2, Section 5.3 and Section 5.4
Generalisability	21	Discuss the generalisability (external validity) of the study results	Section 5.5—most directly applicable to nursing and allied clinical staff in public facilities in the two localities
Other information			
Funding	22	Give the source of funding and the role of the funders for the present study and, if applicable, for the original study on which the present article is based	Funding statement (back matter)

Source: https://www.strobe-statement.org/checklists/ (accessed on 6 August 2026).

## Appendix B. Constructs and Items

**Table A2:** Constructs, Dimensions, and Items.

Constructs	Dimensions	Items
Organisational Culture (OCU)	Involvement (INV)	Decisions are usually made at the level where the best information is available.
		Information is widely shared so that everyone can get the information he or she needs when it’s needed.
		Everyone believes that he or she can have a positive impact.
		Working in this organisation is like being part of a team.
		Our organisation relies on horizontal control and coordination to get work done, rather than hierarchy.
		Teams are the primary building blocks of this organisation.
		Our organisation is continuously improving through learning and internal evaluation.
		Our organisation continuously invests in the skills of employees.
		The capability of people in this organisation is viewed as an important source for long-term success.
	Consistency (CON)	The leaders and managers follow the guidelines that they set for the rest of the organisation.
		There is a clear and consistent set of values in this organisation that governs the way we do business.
		Our organisation has an ethical code that guides our behaviour and tells us right from wrong.
		When disagreements occur, we work hard to achieve solutions that benefit both parties in the disagreement.
		It is easy to reach consensus, even on difficult issues.
		*We often have trouble reaching agreement on key issues.*
		People from different organisational units still share a common perspective.
		It is easy to coordinate projects across functional units in this organisation.
		There is good alignment of goals across levels of this organisation.
	Adaptability (ADA)	Our organisation is very responsive and changes easily.
		Our organisation responds well to changes in the external environment.
		Our organisation continually adopts new and improved ways to do work.
		Customer comments and recommendations often lead to changes in this organisation.
		Customer input directly influences our decisions.
		*The interests of the final customer often get ignored in our decisions.*
		We view failure as an opportunity for learning and improvement.
		Our organisation encourages new initiatives and a spirit of innovation and bold thinking.
		We make certain that we coordinate our actions and efforts between different units in this organisation.
	Mission (MIS)	Our organisation has long-term purpose and direction.
		Our organisation has a clear mission that gives meaning and direction to our work.
		Our organisation has a clear strategy for the future.
		There is widespread agreement about the goals of this organisation.
		Leaders of this organisation set goals that are ambitious, but realistic.
		The leadership has clearly stated the objectives we are trying to meet.
		We have a shared vision of what this organisation will be like in the future.
		Leaders of this organisation have a long-term orientation.
		Our vision creates excitement and motivation for our employees.
Organisational Commitment (OC)		I am willing to perform any duty to remain in this organisation.
		I am willing to work beyond the normal level to succeed in an organisation.
		I feel a strong sense of belonging to our organisation.
		The values our organisation pursues are largely in line with mine.
Knowledge-Sharing Behaviour (KSB)		I share work reports and official documents with members of my organisation more frequently in the future.
		I always provide manuals, methodologies and models for members of my organisation.
		I share my experience or know-how from work with other organisational members more frequently in the future.
		I always provide know-where or know-whom at the request of other organisational members.
		I share expertise from my education or training with other organisational members in a more effective way.
Job Performance (JP)	In-Role Performance (INP)	I fulfil all the responsibilities specified in my job description.
		I consistently meet the formal performance requirements of my job.
		I conscientiously perform tasks that are expected of me.
		I adequately complete all of my assigned duties.
		*I sometimes fail to perform essential duties of my job.*
		*I sometimes neglect aspects of the job that I am obligated to perform.*
	Organisational Citizenship Behaviour towards Organisation (OCBO)	*I sometimes take undeserved or extended work breaks.*
		I adhere to informal organisational rules devised to maintain order.
		I always give advance notice when I am unable to come to work.
		*I sometimes spend a lot of time in personal phone conversations.*
		My attendance at work is above the norm.
		*I sometimes complain about insignificant or minor things at work.*
	Organisational Citizenship Behaviour towards Individuals (OCBI)	I generally help others who have been absent.
		I take a personal interest in the well-being of other employees.
		I generally help others who have heavy workloads.
		I go out of the way to help new employees.
		I generally take time to listen to co-workers’ problems and worries.
		I pass along work-related information to co-workers.
Digital Transformation (DT)		In my organisation, we aim to digitalise everything that can be digitalised.
		In my organisation, we collect large amounts of data from different sources.
		In my organisation, we aim to create more robust networking with digital technologies between the different work processes.
		In my organisation, we aim to enhance an efficient customer interface with digitality.
		In my organisation, we aim at achieving information exchange with digitality.

Note. Items in italics denote negatively phrased items in the survey. To ensure all items moved in the same direction, responses were reversed-scored before statistical analysis.
